# Energy-Efficient ZigBee-Based Wireless Sensor Network for Track Bicycle Performance Monitoring

**DOI:** 10.3390/s140815573

**Published:** 2014-08-22

**Authors:** Sadik K. Gharghan, Rosdiadee Nordin, Mahamod Ismail

**Affiliations:** Department of Electrical, Electronic and System Engineering, Faculty of Engineering and Built Environment, Universiti Kebangsaan Malaysia, UKM Bangi, 43600 Selangor, Malaysia; E-Mails: adee@eng.ukm.my (R.N.); mahamod@eng.ukm.my (M.I.)

**Keywords:** cycling, power consumption, wireless sensor network, ZigBee

## Abstract

In a wireless sensor network (WSN), saving power is a vital requirement. In this paper, a simple point-to-point bike WSN was considered. The data of bike parameters, speed and cadence, were monitored and transmitted via a wireless communication based on the ZigBee protocol. Since the bike parameters are monitored and transmitted on every bike wheel rotation, this means the sensor node does not sleep for a long time, causing power consumption to rise. Therefore, a newly proposed algorithm, known as the Redundancy and Converged Data (RCD) algorithm, was implemented for this application to put the sensor node into sleep mode while maintaining the performance measurements. This is achieved by minimizing the data packets transmitted as much as possible and fusing the data of speed and cadence by utilizing the correlation measurements between them to minimize the number of sensor nodes in the network to one node, which results in reduced power consumption, cost, and size, in addition to simpler hardware implementation. Execution of the proposed RCD algorithm shows that this approach can reduce the current consumption to 1.69 mA, and save 95% of the sensor node energy. Also, the comparison results with different wireless standard technologies demonstrate minimal current consumption in the sensor node.

## 1. Introduction

Wireless sensor networks (WSNs) have many uses in tracking and monitoring [1], where they have attracted more attention in recent years. The applications of WSN can be classified into industrial, biomedical [2], environmental, military, agricultural [3], domestic, and commercial fields [4]. In the last few years, WSNs have received considerable attention in sports applications for monitoring athletes' performance during training sessions as well as in international competitions [5–8]. Cycling is one of the sports that has recently attracted significant attention in this respect, and WSNs are widely used for monitoring the physiological and biomechanical parameters of the athlete and bike, respectively, in order to assess cycling performance. Cycling performance can be monitored by using unobtrusive sensor nodes; these nodes comprise different components such as a sensor, data processor, transceiver module, and power unit.

The radio transmission in a WSN is considered the main power consumer and uses considerable power [9]. Prolonging the battery lifetime of the sensor node is accomplished by minimizing the data transmission and reception. The sensor node is battery powered, which is a major drawback which limits the lifetime of the entire WSN [10]. The battery often requires recharging or replacement from time to time due to its finite power capacity. One of the important issues for enhancing the performance of WSNs is improving the performance of the sensor node. Therefore, reducing power consumption in the sensor node is an urgent demand for WSNs.

Many researchers have looked at how to reduce the power consumption of WSNs based on various factors that affect energy management. An energy-efficient WSN based on a CC1101 RF communication module and MSP430F149 microcontroller platform [11] was assembled to minimize energy consumption at two levels; the network level through adaptive network topology configuration and at the sensor node level by using adaptive transmission power according to the distance between two nodes, and a sleep/wake-up scheme. The application of that platform was prompted by greenhouse temperature management, in which the temperature is measured by the sensor nodes. The temperature varies slowly, so not all the sensor nodes are required to stay awake. The sensor node spends most of the time in sleep mode [11]. Energy saving was introduced in [12] by adopting two mechanisms, namely RF communication power control and node sleep scheduling. The sensor nodes constantly sense and sends data about the ambient temperature to the sink node. The energy saving was improved based on a test bed network to 60% and 16.7% by applying a suitable sleep/wake-up mechanism and power control scheme, respectively. In another research [13] all ZigBee nodes in the network used a proposed sleeping technique at the application layer. The network was divided into smaller groups to reduce packet collision and latency. The proposed sleeping technique resulted in about 90% energy savings for a WSN consisting of 20 nodes per group. These strategies used in previous research are not useful in bike sensor nodes because the speed and cadence data are updated every second or every wheel rotation on the one hand and on the other hand, the distance between the bike speed and cadence sensor node and coordinator node is constant.

A number of works have been carried out related to the utilization of environmental and mechanical sources to harvest energy by supply or charge the sensor nodes to extend the WSN lifetime. These environmental sources are available in many forms, including thermal energy, electromagnetic waves, and sunlight [14,15], while the mechanical energy sources are generated when a sensor node is subjected to some mechanical deformation or movement and modified into electrical energy by several methods, including electromagnetic, electrostatic, and vibration based on piezoelectric conversion [16,17]. The aforementioned of the two energy sources can provide an almost infinite lifetime for a sensor node and can produce up to tens of mW of power [18,19], depending on transducer size and type. However, these alternatives energy sources cannot be applied to a track bike sensor node because it will increase the aerodynamic and the rolling resistance, for example when using a bicycle dynamo as a mechanical source for energy harvesting. These factors lead to a reduction in bike speed, increase the weight of the bike and induce fatigue to the athlete during cycling, which is critical in a competitive event.

The use of wireless sensor nodes in bike performance monitoring is somewhat different from other applications of WSNs such as temperature monitoring, where the sensor node sleeps most of the time. In the latter case, the collected data are transmitted quickly by the sensor node through a wireless link in a short time, and when the transmission is complete the sensor node returns to sleep mode. However, in a bike WSN, the data of bike parameters are transmitted directly from a sensor node to the central node at least every bike wheel rotation [20] or every second or half second, so the sensor nodes in the bike WSN cannot remain in sleep mode for a long time. In such a case, a new method must adopted to reduce the power consumption in the sensor node without affecting the performance of the measurement parameters. To prolong the wireless sensor battery life, a robust Redundancy and Converged Data (RCD) algorithm is proposed. This algorithm is specially designed to minimize the amount of data transmissions and put the sensor node into sleep mode when the bike speed and cadence data are redundant or converge. The RCD algorithm was designed to provide a solution to the power consumption problem in a bike sensor node in the following ways: (i) data packet transmission is minimized based on a particular strategy; (ii) sensor nodes in the bike network can be reduced to one based on measurement correlation; and (iii) mathematical calculation of the cadence can be done in a central (coordinator) node based on the natural correlation between speed and cadence to reduce the data processing time in the sensor node.

The rest of the paper is ordered as follows: both the bike speed and cadence will be modelled mathematically in Section 2. The implementation of the entire framework will be explained in Section 3. The study assumptions will be presented in Section 4, and the framework setup based on XCT-U software will be demonstrated in Section 5. The RCD algorithm will be described in Section 6, while the power consumption and lifetime of the sensor node will be estimated and calculated in Sections 7 and 8, respectively, and the results compared with standard wireless technologies and previous work in Section 9. Finally, conclusions will be drawn in Section 10.

## 2. Bike Speed and Cadence Module

The bike speed and cadence can be measured due to the known laws of physics. The bike angular velocity *Ω* can be measured as follows:

(1) $$\Omega =\frac{2\pi }{\Delta t}$$

where Δ*t* is the time elapsed for one bike wheel rotation in seconds.

The relationship between wheel angular velocity *Ω* and wheel linear speed υ*_b_* (bike speed in kilometers per hour) is given by:

(2) $$\upsilon_{b}=3.6\Omega \cdot r$$

where *r* is the track bike wheel radius in meters and 3.6 is the unit transformation of meters per second into kilometers per hour.

The wheel angular velocity *Ω* is related to the pedal angular velocity *ω* (the cadence CAD in revolution per minute (RPM)) through the gear ratio *F/R*. It can be modelled by Equation (3) [21]:

(3) Ω=ω·(F/R)·(2π/60)

where *F* is the teeth number on the front sprocket (44 teeth), *R* is the teeth number on the rear sprocket (16 teeth for a track bike type), and 2π/60 is the unit transformation of RPM to radians per second.

Combining and rearranging Equations (2) and (3), the cadence pedal bike (*ω*) in RPM can be formulated in relation to linear bike speed as follows:

(4) $$\omega =\text{Cadence}=\left(\upsilon_{b}/3.6r\right)\;\cdot \;\left(R/F\right)\;\cdot \;(60/2\pi )$$

where *F*, *R*, and *r* are constant values of the track bike.

Therefore, using Equations (2) and (4), the track bike speed and cadence can be measured by using one sensor node consists of a magnetic sensor, microcontroller, ZigBee module, and a power unit. This will reduce the power consumption, cost, size, and hardware complexity. On the contrary, SRM [22], POLAR [23], and some of the other manufacturers use two separate sensor nodes for speed and cadence measurement.

## 3. Framework Implementation

**Figure 1.**
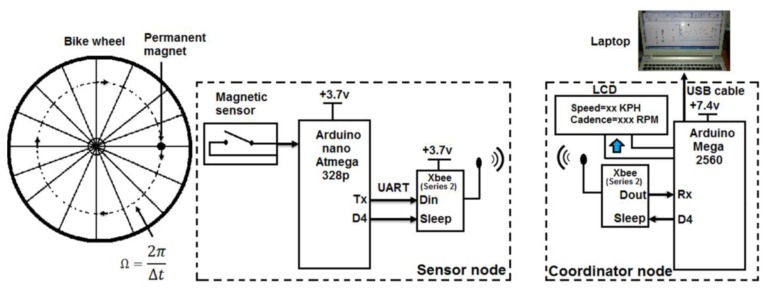
Block diagram of the whole framework of bike speed and cadence measurement.

**Figure 2.**
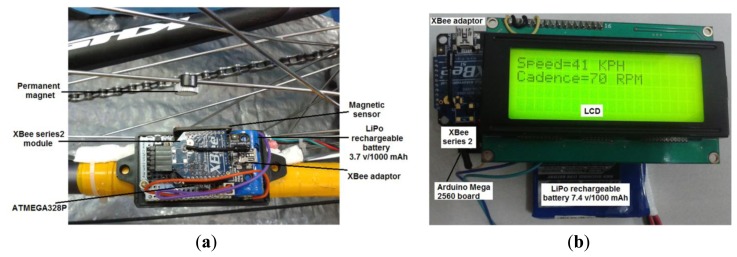
A snapshot of the full framework: (**a**) sensor node with its parts mounted on bike chainstays and (**b**) the coordinator node with its parts mounted on the seat post under the rider seat (saddle).

The entire framework of bike speed and cadence data measurement and transfer consists of two nodes; the sensor node and coordinator node. The sensor node has four basic components: a magnetic sensor with a small permanent magnet, an ATmega 328P microcontroller as a Barebone, a ZigBee module (XBee series 2), and a power unit (LiPo rechargeable battery 3.7 v/1000 mAh). The coordinator also has four components: an Arduino Mega board based on the Mega 2560 microcontroller, liquid crystal display (LCD), ZigBee module (XBee series 2), and power unit (LiPo rechargeable battery 7.4 v/1000 mAh). The entire framework is shown in the block diagram in Figure 1 and a snapshot in Figure 2. For future work, the bicycle parameters will be wirelessly transferred to the coach's laptop to monitor the speed and cadence of the bike. The use of smartphone is not encouraged in high-performance sports, which include track cycling events (typically held in a velodrome) due to weight savings and to avoid unnecessary distraction to the athlete. In this paper, reduced power consumption in the sensor node will be considered. All components will be described in more detail in the following subsection.

### 3.1. Magnetic Sensor

A magnetic sensor is employed to pass a digital signal to the ATmega 328P microcontroller. These signals represent the time interval of wheel rotations. A magnetic sensor (reed switch) and a permanent magnet form the sensing part of the framework. The magnetic sensor is mounted on the chainstay of the rear wheel, and a permanent magnet is fixed on one spoke of the wheel. When the bike wheel begins to spin, the permanent magnet passes through the magnetic sensor and the reed switch is closed. The time elapsed for one wheel rotation can be measured by a microcontroller based on the mathematical representation in Equation (1) in Section 2. Therefore, the linear speed of the bike can be calculated in kilometers per hour (km/h) or miles per hour (mph) based on Equation (2) in Section 2, where the wheel radius is equal to 0.3683 m for the track bike model adapted in this experiment. As a result, the bike cadence can be calculated in RPM in a microcontroller of the sensor node or in the coordinator node based on the speed data by applying Equation (4) in Section 2. In this study, both speed and cadence were calculated in the sensor node, since the focus is on the evaluation of sensor node power consumption. In addition, the SRM system [22], used for comparison purposes also measures and sends both cadence and speed data separately. The Polar system [23], as well with other commercial bike power meters in the market oparete similarly. It is expected that the cadence calculation in the coordinator node can further improve the energy savings, which is a potential extension to the current work.

### 3.2. Microcontroller

The microcontroller in a sensor node based on the ATmega 328P platform is used for data acquisition of bike speed and cadence parameters. This microcontroller is small and it is targeted at low power consumption applications [24,25], so it is extremely suitable for this application as a core of the sensor node. The microcontroller was programmed using the C language [26], and an RCD algorithm was built inside this microcontroller. The RCD algorithm will be explained in detail in a later section. The RCD algorithm acquires the digital signal from the magnetic sensor in the case of sensor firing as part of this task. It processes this signal and converts it into data, which represent the bike speed. These data are transmitted as data frames at a maximum baud rate of 115,200 bps (86.6 μs for one frame consisting of 10 bits) to ZigBee via the microcontroller serial protocol. A high baud rate of 115,200 bps was selected in order to decrease the frame transmission time, thereby reducing the power consumption of the microcontroller, as will be seen in a subsequent section.

### 3.3. ZigBee Wireless Module

The ZigBee is an independent module; it is a cost-effective component that uses 2.4 GHz radio frequency (RF) [27] with data throughput of up to 250 kbps [28] for transferring data between ZigBee modules. The ZigBee uses the IEEE 802.15.4 protocol known as a Low-Rate Wireless Personal Area Network (LR-WPAN). This protocol implements media access control, addressing, error detection, and acknowledgements and retries to ensure data integrity and delivery [29]. The ZigBee module is small in size and can be included in different sensor nodes. In addition, it has been designed to operate with low power consumption based on a unique configuration of sleep mode and to transfer data speedily between nodes in a WSN for use in control systems and monitoring [27,29].

Although the ZigBee does not have a microcontroller, it does have a finite amount of self-processing capability which it can use to control itself, such as sleep mode for prolonging battery life [27]. Therefore, in this paper, it is used with an external small microcontroller to perform a particular algorithm to interpret or carry out some mathematical calculations that cannot be performed by ZigBee alone. The wireless connection of this framework consists of two ZigBee nodes (XBee series 2): One acts as a sensor node to transmit the data of bike speed and cadence while the other acts as a coordinator node to receive this data. The X-CTU software from DIGI International [30] was used to configure the sensor node with the microcontroller and a coordinator node to establish a communication protocol between the two ZigBee nodes.

## 4. Research Assumptions

- The sensor node does not require large-scale data storage because the data are transmitted in real time with small amounts of digital data from the bicycles, approximately at 20 bits for speed and cadence data, 10 bits per sample for each one and 5 Kbyte for microcontroller program.
- The data are transmitted every second in the case in which the RCD algorithm is not applied.
- There is no re-transmission of data in the case of data packet loss because the data is continuously updated.
- The difference between the preceding and subsequent data speeds is that for accuracy purpose, it is limited to 1 km/h (*i.e.*, ±5% to ±10%, corresponding to 20 to 10 km/h).
- The initial conditions for speed and cadence parameters are assumed to be random.
- For clarity of the results presentation, a time window of 320 s will be considered in this experiment. This is more than the time required to complete 16 laps in a real track competition, whereby each lap with an average of 15 s, *i.e.*, total of 240 s in 16 laps [31].

## 5. Experimental Setup

The network of this system was composed of simple point-to-point networks, and two ZigBee (XBee series 2) nodes were used. One module acts as a sensor node and is mounted on the bike chainstay to transmit the bike speed and cadence data. The other node is the coordinator node, which is connected to a laptop to display the received data in real time based on X-CTU software on one hand and to display the received data on the LCD to be viewed by the cyclist on the other hand. Table 1 shows the configuration of both the sensor and the coordinator node based on X-CTU software. To ensure communication between the two ZigBee modules in the network, the network ID, operating channel (CH), and baud rate (BD) should be the same. The Serial Number High (SH) of the sensor node must be the same as the Destination Address High (DH) of the coordinator node and vice versa, and the Serial Number Low (SL) of the sensor node must be the same as the Destination Address Low (DL) of the coordinator node and vice versa. The transmitted power is configured to the low RF power setting (*i.e.*, −10 dBm) in order to reduce power consumption in the sensor network. In addition, the distance between the sensor node and coordinator nodes is very short (≈ 1 m), so there is no need to transmit at higher RF power.

In WSN applications, most of the nodes in the network are battery powered; therefore, the coordinator node is obliged to wake up very frequently to process several functions such as hop routing, acknowledging signals and receiving data. Other nodes in the network remain asleep and perform cyclic checks on whether they need to wake up and become active. However, in some applications the coordinator node may enter sleep mode [32], so in this application, to improve the power consumption of the whole network the microcontroller in the coordinator itself can be configured or programmed to enter sleep mode when no data are received. This enables essential power saving in the coordinator node along with the sensor node.

## 6. RCD Algorithm Description

The proposed RCD algorithm is given in Algorithm 1, was designed to perform the following tasks:

- Minimization of the data packets transmitted to as low as possible based on the difference between preceding and subsequent data. If the speed difference is less than or equal to 1 km/h (the speed difference = old speed minus new speed ≤1 km/h), the ZigBee will be in sleep mode for a significant time because the bike speed is not changing rapidly, especially in international track competitions, whereby the average bike speed is 60 km/h [31,33]. In this case, the transmitted packets, which represent bike speed will be less. When the speed difference is greater than 1 km/h, the ZigBee wakes up and data are transmitted every second.
- Reduction of the number of sensor nodes of the framework to one node depends on fusing data and the correlation measurements between bike speed and cadence. This will reduce power consumption, cost, size, and enable simpler hardware implementation in the whole framework. This leads to lower data packet transmission.
- Using the maximum baud rate of microcontroller serial protocol to reduce the time elapsed in the active state, thereby reducing power consumption.
- Putting some functions of the microcontroller in sleep mode when there is no sensor firing or between sensing events in order to reduce the power consumption in processing unit. The sleep modes were used as much as possible and the sleep mode of the microcontroller was selected so that as few as possible of the device's functions are operating [34]. Therefore, the Timer 1 register which already exists inside the microcontroller will keep running during the sleep mode and it is able to measure the time elapsed for wheel rotation. The processing unit of microcontroller wakes up after sleeping and resumes execution through global interrupt when the magnetic sensor sense the bike wheel rotation and send logic ONE to the pin2 of microcontroller to active the interrupt function of the microcontroller.

The parameters of the proposed RCD algorithm are defined in Table 2 as follows:

**Algorithm 1: Pseudocode for the Proposed RCD Algorithm**
1:New_speed = 0; // Initial condition set to zero2:**if** Timer 1 of the microcontroller is interrupted at 1 ms {3:Reed_switch = **digitalRead** (Magnetic sensor) // Check sensor date to measure new speed4:**if** (Reed_switch ==1) { // *If magnetic sensor is closed*5:Wake up the microcontroller //The magnetic sensor sends logic ONE to interrupt pin2 of // the microcontroller to active the interrupt function.6:**if** (Counter == 0) { //Minimum time 200ms for debouncing the magnetic sensor has passed7:Calculate bike **Speed** according to Equation (2);8:Calculate bike **Cadence** according to Equation (4);9:Speed_difference = Speed – New _speed;10:New_speed = Speed;11:Timer = 0; // Reset timer12:Counter =200; // Minimum time required for debouncing (200ms)13:}14:**else** {15:**if** (Counter > 0) { // Do not let counter go to negative16:Counter = Counter-1; // Decrement Counter17:}18:}19:}20:**else** { // *Magnetic sensor is open*21:**if** (Counter > 0) { // Do not let counter go to negative22:Counter =Counter-1; // Decrement Counter23:}24:}25:**if** (timer > 2000) { // No new pulses from Magnetic sensor (the bike wheel does not rotate)26:**Speed** = 0;27:**Cadence** = 0;28:}29:**else** {30:Timer = Timer+1; // Increment time31:}32:}33:**void** Pin2_microcontroller_interrupt **(void)** { // Microcontroller interrupt function34:**detachInterrupt (1)**; // Detach the interrupts to stop it from continuing firing while the // interrupt pin2 (INT 0) is High35:}36:**void** enterSleep **(void)** { // Bring the microcontroller into sleep mode37:**attachInterrupt** (0, Pin2_microcontroller_interrupt, RISING); // Interrupt occur when the // voltage rises from 0 to 3.7 volts on pin2 (INT 0) of the microcontroller38:delay (100);39:**set_sleep_mode** (SLEEP_MODE_PWR_SAVE); // Setting sleep mode type of the // microcontroller40:**sleep_enable ();** // Microcontroller sleep enable41:}42:**void** loop () { // main loop43:**while** (fabs (Speed_difference) > 1) { // Stay in the loop when the absolute value of the // Speed_difference is greater than 1km/h otherwise out of the loop44:**while** (Reed_switch==1) { // Stay in the loop when the magnetic sensor is closed otherwise // out of the loop45:**digitalWrite** (Xbee, LOW); // The microcontroller assert logic ZERO to pin 9 // (Hibernate pin) of the Xbee module to wake up the Xbee series 2 module46:**Serial. println** (Speed, DEC); // Transmit Speed data to the coordinator node47:delay (500);48:**Serial. println** (Cadence, DEC); // Transmit Cadence data to the coordinator node49:delay (500);50:}51:}52:**digitalWrite** (Xbee, HIGH); // The microcontroller assert logic ONE to the pin 9 // (Hibernate pin) of the Xbee module to put the XBee series 2 module in sleep mode53:**enterSleep ()**; // The microcontroller enter sleep mode when the bike wheel does not rotate54:} // End of the main loop

## 7. Sensor Node Power Consumption and Lifetime Estimation

The lifetime of the WSN is defined as the time elapsed from initial dissemination to the instant when the connection reaches a preset threshold level [35]. In this paper, the lifetime of the sensor node may be defined as the time from the start of the sensor node's sensing event until the battery power runs out. The lifetime of the sensor node depends on the current consumption of the main three components in the sensor node: Magnetic sensor, microcontroller, and RF communication (ZigBee module). When these power consumers added together, we determine the expression of full current consumption in the sensor node as in Equation (5):

(5) $$I_{\text{sensor node}}=I_{ms}+I_{\mu c}+I_{xb}$$

where *I_ms_* is the current consumption of the magnetic sensor, *I_μc_* current consumption of the microcontroller, and *I_xb_* current consumed by RF module (ZigBee).

In order to estimate the power consumption in the sensor node, current consumption measurements were done for various components in the sensor node by using digital Ampere meter device. So, the current consumption was formulated for two cases. In the first case, the current consumption in the sensor node (*I_Sensor node_*) was estimated without applying the RCD algorithm and can be formulated in Equation (6). In the second case; the RCD algorithm was applied. So the estimation of current consumption in the sensor node can be formulated in Equation (7). The parameters in Equations (6) and (7) is described in Table 3, and Table 4 as measured values for current consumption and time elapsed for each operation, respectively:

(6) $$I_{\text{Active}-\text{idle}}=\frac{\left[\left(I_{\text{msa}}\times t_{\text{msa}}+I_{\text{msi}}\times t_{\text{msi}}\right)+\left(I_{\mu ca}\times t_{\mu ca}+I_{\mu ci}\times t_{\mu ci}\right)+\left(I_{\text{xba}}\times t_{\text{xba}}+I_{\text{xbi}}\times t_{\text{xbi}}\right)\right]}{\text{Total}\;\text{period}\;\text{of}\;\text{test}\;\text{time}}$$

(7) $$I_{\text{Active}-\text{sleep}}=\frac{\left[\left(I_{\text{msa}}\times t_{\text{msa}}+I_{\text{msi}}\times t_{\text{msi}}\right)+\left(I_{\mu ca}\times t_{\mu ca}+I_{\mu cs}\times t_{\mu cs}\right)+\left(I_{\text{xba}}\times t_{\text{xba}}+I_{\text{xbs}}\times t_{\text{xbs}}\right)\right]}{\text{Total}\;\text{period}\;\text{of}\;\text{test}\;\text{time}}$$

Equation (6) is somewhat similar to Equation (7), except for the presence of idle state for the microcontroller and ZigBee in Equation (6) and its absence in Equation (7). On the other hand, the sleep state is present for the microcontroller and ZigBee in Equation (7) and absent in Equation (6). This is due to the application or non-application of the RCD algorithm. The sensor node lifetime (*L*) can be estimated as in (8) [35,36]:

(8) $$L=\frac{I_{\text{Supply}}}{I_{\text{sensor node}}}$$

where *I_Supply_* is the initial current capacity of the sensor node battery. Since the power supply based on a LiPo rechargeable battery 3.7v/1000 mAh in this experiment, the calculation of current consumption and lifetime of the sensor node will be achieved in both cases with and without the RCD algorithm in the next section.

## 8. Power Consumption and Lifetime Calculation Results

The power consumption and lifetime of the sensor node will be estimated with and without applying an RCD algorithm as follows:

### 8.1. Without Applying the RCD Algorithm

**Figure 3.**
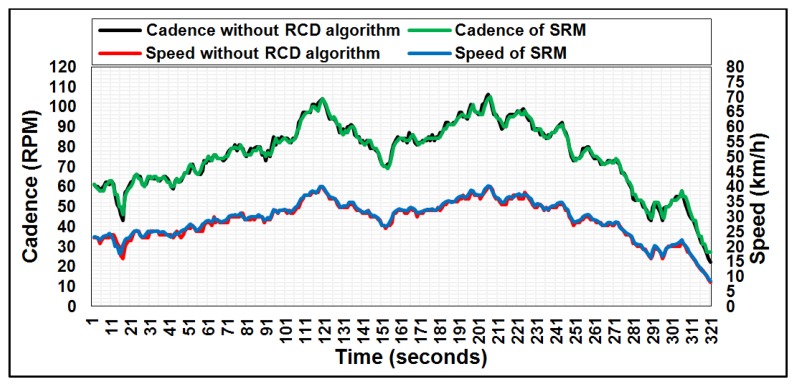
Real time measurements of bike speed and cadence for the proposed system and the SRM system.

The results were in a harmony with the research assumptions. Once the framework setup was complete, the cyclist began pedaling and the experiment was conducted multiple times for 15 min in indoor conditions for a track bike fixed on a Tacx Cycle trainer. The sensor node began to transmit the data of bike speed and cadence every second. The data were received by the coordinator node and analyzed by the Arduino Mega 2560 microcontroller. The data obtained by the microcontroller were sent to the laptop via a USB port and were recorded in Parallax Microcontroller Data Acquisition for Excel (PLX-DAQ), a free-license software designed by the company Parallax [37]. The received data on speed and cadence were plotted as a continuous signal with respect to the total test time, as shown in Figure 3. The proposed system without RCD algorithm is compared with SRM [22], which is commonly used by the professional track cyclist in international tournaments [38]. The speed and cadence data for the two systems were reported every one second, as shown in Figure 3. From the graph, the speed and cadence data are highly consistent. The sensor node lifetime of the mathematical model in Equation (8) can be estimated based on the current consumption measurement parameters of Equation (6). The current consumption can be calculated for all components in the sensor node as follows:

#### 8.1.1. Current Consumption Calculation for Magnetic Sensor

The magnetic sensor is a magnetic switch (reed switch). When the permanent magnet moves near to the magnetic sensor, the magnetic sensor is closed and consumes 0.5 mA, while the microcontroller reads a logic ‘ONE’. When the permanent magnet moves away from the magnetic sensor, the magnetic sensor is open (zero current consumption) and the microcontroller will read a logic ‘ZERO’ due to the internal pull-up resistor of the microcontroller [39]. Therefore, current consumption of the magnetic sensor occurs only when the permanent magnet passes through the magnetic sensor and is zero during the rest of the time. The average bike speed in this experiment is 29 km/h, which means almost 300 ms of time elapses during one wheel rotation, based on Equations (1) and (2). This means that the frequency of wheel rotation is 3.33 Hz. Therefore, the bike wheel will rotate 1065 times during the test time. For example, if the maximum active passing time (sensor firing) is 30 ms (10% of the time elapsed during one wheel rotation) and the rest of the time is idle time (zero current consumption). This means the total sensing time is equal to 32 s (1065 × 30 ms) from the test time and the current consumption amounts to 0.05 mA according to the first term of the right part of the Equation (6).

#### 8.1.2. Current Consumption Calculation for Microcontroller

The ATmega 328P microcontroller sends data frames, which represent the data of bike speed and cadence, to the ZigBee every second. As mentioned previously, the average bike speed is 29 km/h, and therefore the total active time of the microcontroller is 32 s, corresponding to magnetic sensor firing, and the idle time was 288 s during the test time. The current consumption of the microcontroller may be calculated by substituting the related values in Tables 3 and 4 in the second term of the right part of the Equation (6). Therefore, the total current consumption of the microcontroller is 9.55 mA.

#### 8.1.3. Current Consumption Calculation for ZigBee (XBee series 2)

The 802.15.4 standard allows a maximum packet size of 127 bytes, including payload [40] and a data rate of 250 Kbps at RF 2.4 GHz, which means 4 μs transmission time for one bit or 32 μs for one byte. This means that the time on the air is 4.064 ms for one packet with a size of 127 bytes, where CSMA-CA and retry time are neglected because the application uses simplex communication type. Therefore, the total active time required to transmit data is 1.3 seconds (320 × 4.064 ms) and the idle time is 318.7 seconds during the total test time. In this example, the gross current consumption of the ZigBee of 28 mA was obtained by applying the third term of the right part of the Equation (6). The gross current consumption of the sensor node according to Equation (6) is 37.6 mA supplied by a LiPo rechargeable battery 3.7 v/1,000 mAh. Therefore, the lifetime of the sensor node is 27 h, according to Equation (8).

### 8.2. Application of RCD Algorithm

**Figure 4.**
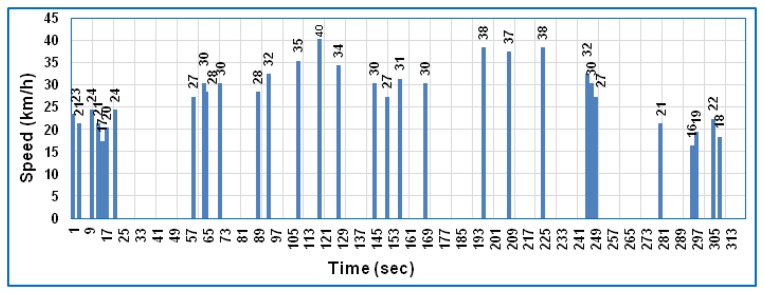
The bike speed in the case of applying the RCD algorithm.

**Figure 5.**
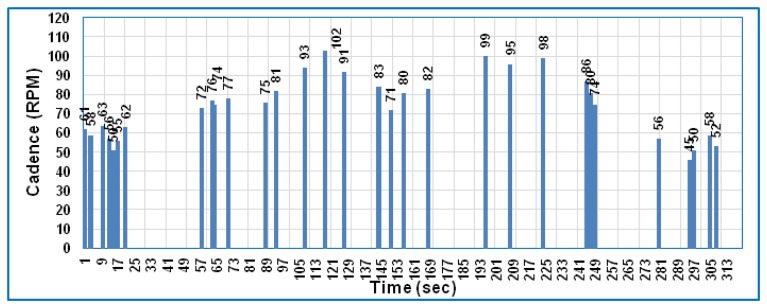
The bike cadence in the case of applying the RCD algorithm.

When the RCD algorithm is applied to the framework, as explained previously in Section 6, the received data can be collected and analyzed as shown in Figures 4 and 5 for both speed and cadence, respectively, based on PLX-DAQ software.

These two figures show the received data only at a particular time depending on the difference between the preceding and subsequent data of bike speed and cadence, which means the ZigBee is active only at this particular time and sleeps during the time slot. Comparing Figure 3 with Figures 4 and 5 for speed and cadence, respectively, speed and cadencedata are transmitted every second in Figure 3, which means the sensor node consumes more power; whereas in Figures 4 and 5, the data are transmitted at a particular time depending on the RCD algorithm. Therefore, the percentage representation of data transmission is noted that 10% of the total test time compared to the standard case (without the RCD algorithm). Hence, the current consumption of the three components in the sensor node can be calculated in the same way as explained previously in Section 8.1, bearing in mind that the sleep mode is available and the idle state disappears in this case (with the RCD algorithm).

The current consumption of the magnetic sensor is the same as in the first case (without applying the RCD algorithm), *i.e.*, it is 0.05 mA according to the first term of the right part of Equation (7). However, for the microcontroller and ZigBee the current consumption is different due to the sleep mode, which significantly reduces the power consumption of the sensor node as we will note later, so the current consumption of these two components can be estimated as follows.

#### 8.2.1. Current Consumption Calculation for Microcontroller

The ATmega 328P microcontroller cannot sleep for a long time, because the bike speed and cadence data are updated every second and sent to the ZigBee module depending on the speed difference. Therefore, the microcontroller can sleep only in the time elapsed in one rotation, which depends on the cyclist's speed. As mentioned previously, the average bike speed is 29 km/h, which means the total microcontroller active time was 32 s and the total microcontroller sleep time was 288 s during the test time. The current consumption of the ATmega 328P microcontroller is equal to 10 mA in active mode and 0.65 mA in sleep mode, as listed in Table 3 based on digital Amper meter reading. Therefore, the total current consumption of the microcontroller of 1.58 mA was obtained by applying the second term of the right part of the Equation (7).

#### 8.2.2. Current Consumption Calculation for ZigBee (Xbee Series 2)

The ZigBee module transmits data only when the speed difference is greater than 1 km/h; otherwise, the ZigBee enters sleep mode. Figures 4 and 5 show the bike speed and cadence data, respectively, which are transmitted only 31 times during the total test time. The time required for transmitting one full packet (127 byte) is 4.064 ms. Therefore, the total active time required to transmit the data 31 times was 0.126 s and the sleep time was 319.874 s during the total test time. In this example, the gross current consumption of the ZigBee of 0.0617 mA was obtained by applying the third term of the right part of the Equation (7). According to Equation (7), the total current consumption of a sensor node obtained by applying the RCD algorithm is 1.69 mA. When Equation (8) is applied with the same battery capacity as in the first case, the sensor node lifetime becomes 590 h.

Execution of the RCD algorithm shows that this approach can reduce the current consumption to 1.69 mA at a sensor node. This will prolong the battery life to 590 h based on a lithium polymer (LiPo) rechargeable battery 3.7 v/1000 mAh. This leads to 95% of energy being saved in comparison to tests without the RCD algorithm, making the current consumption 37.6 mA and the sensor nodes' battery life 27 h.

**Figure 6.**
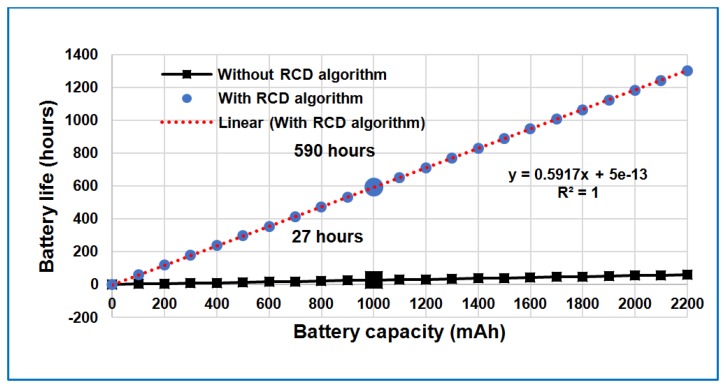
Expected battery life *versus* usable battery capacity in the sensor node.

The estimated battery lifetime for a particular battery capacity at this sensor node current usage is illustrated by the linear relationship in Figure 6. This figure also shows improvement of current consumption in the sensor node when the RCD algorithm is applied.

## 9. Comparison of Results

**Figure 7.**
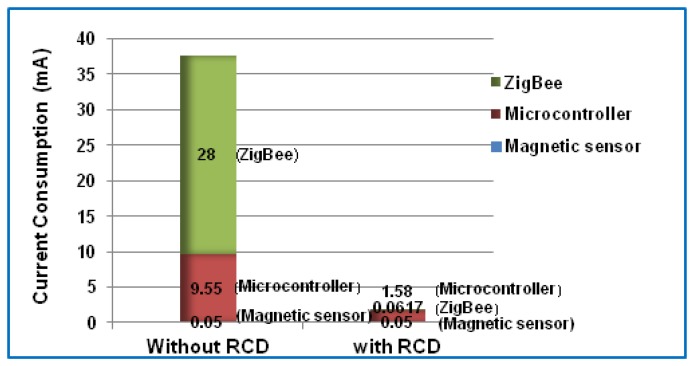
The current consumption of components of the sensor node with and without applying the RCD algorithm.

The current consumption was measured for various components of the sensor node based on the digital Amper meter device. The current consumption of each part of the sensor node was compared to two cases with and without applying the RCD algorithm as shown in the bar chart in Figure 7. As can be seen from this figure, when the first case (without applying the RCD algorithm) is discussed the RF module (ZigBee) consumes more power of the sensor node. This corresponds with the findings of previous works [2,41–45]. Moreover, the microcontroller comes in the second order for power consumption. Also, Figure 7 shows the development of power consumption in both the microcontroller and ZigBee modules after applying the RCD algorithm. However, in this case the microcontroller still consume more current than Zigbee module in the sensor node.

**Figure 8.**
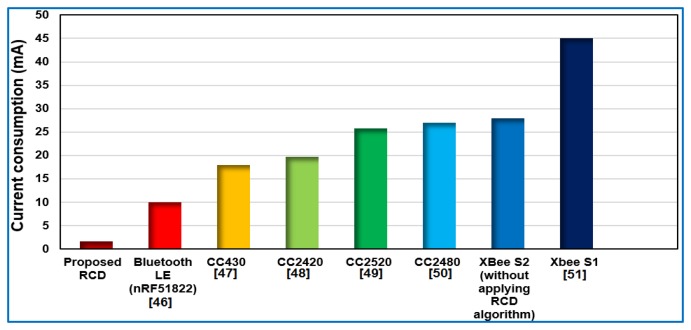
The current consumption of the sensor node compared with different wireless standard technologies.

Furthermore, the power consumption of the sensor node presented in this work can be compared with other works and different wireless standard technologies [46–51], as shown in the bar chart in Figure 8. This figure shows significant current savings when the proposed RCD algorithm is applied.

## 10. Concluding Remarks

The most significant challenge in a WSN for the described application (track cycling) is minimizing power consumption in the sensor nodes due to miniaturization of the electronic devices and the light weight requirement for track cycling. In this paper, energy efficiency of the sensor node in a bike WSN was achieved based on a proposed RCD algorithm for bike speed and cadence data transmission. This algorithm introduced the solution of power consumption for this application by putting the ZigBee into sleep mode for redundant and converged data, using the correlation between bike speed and cadence and reducing the number of sensor nodes in the bike WSN while putting the microcontroller in sleep mode when there was no sensor firing.

The framework utilizes a low power ATmega 328P microcontroller, a magnetic sensor with a small permanent magnet, and a ZigBee wireless module to enhance the power consumption in the sensor node. The gross current consumption of the sensor node has been reduced to 1.69 mA, which will prolong the battery lifetime of 590 h and save about 95% of the battery power of the sensor node. The power consumption of the sensor node in this application depended on the application of the RCD algorithm. The RCD algorithm is based on the bike speed, while bike speed may be constant or varied for certain periods depending on the cyclist's actions, so the power consumption may be increased or decreased slightly.

From this research, it can be noted that the greatest amount of power is dissipated in the microcontroller compared to the Zigbee protocol. This was demonstrated clearly when the RCD algorithm was applied, whereby the sensor firing frequency can be reduced to every two seconds to keep the microcontroller asleep for a longer duration. This significantly improves the power consumption of the microcontroller to half, which means the power consumption of the whole bike WSN will be reduced. The comparison results with different wireless standard technologies and previous work showed that the sensor node for bike WSN can highly benefit from the use of RCD algorithms to achieve significant power savings. Therefore, the RCD algorithm can be used for track cycling applications to save more power with extended battery life.

## Figures and Tables

**Table 1.** Configuration of sensor and coordinator nodes based on X-CTU software.

Parameter	Value & Unit
Operating channel (CH)	2.415 GHz
Baud rate (BD) bps	115,200 bps
Data bits	8
Stop bits	1
Parity	None
Power level (PL)	−10 dBm
Sleep mode (SM)	Pin hibernate

**Table 2.** The definition of the parameters in RCD algorithm.

Parameters	Description
Timer 1	16-bit timer Register built inside the microcontroller Atmega 328p, used to calculate the time between two pulses of sensor firing
Reed_switch	Represents the magnetic sensor
New_speed	Captures the updated value of speed, the initial condition set to zero
Counter	Keeps-minimum time (in ms) of one wheel rotation (for debuncing)
Timer	Measurement of the time elapsed for wheel rotation, the initial condition set to zero
Pin2_microcontroller_interrupt	Microcontroller sleep function
fabs	Instruction in C, takes the absolute value of a float
Xbee	Xbee series 2 module

**Table 3.** Current consumption of various components in the sensor node associated to Equations (6) and (7).

Parameters		Operation Case	Current Consumption (mA)
**Magnetic sensor**	*I_msa_*	Sensor firing (active)	0.5
	*I_msi_*	Idle	0

**Microcontroller (processing)**	*I_μca_*	Active	10
	*I_μci_*	Idle	9.5
	*I_μcs_*	Sleep	0.65

**ZigBee module (radio link)**	*I_xba_*	Active	30
	*I_xbi_*	Idle	28
	*I_xbs_*	Sleep	0.05

**Table 4.** Time elapsed of different components in the sensor node related to Equations (6) and (7).

Parameters		Case of Operation	Time Elapsed of Each Operation (s)	

			Without RCD Algorithm	With RCD Algorithm
**Magnetic sensor**	*t_msa_*	Sensor firing (active)	32	32
	*t_msi_*	Idle	288	288

**Microcontroller(processing)**	*t_μca_*	Active	32	32
	*t_μci_*	Idle	288	N/A
	*t_μcs_*	Sleep	N/A	288

**ZigBee module (Radio link)**	*t_xba_*	Active	1.3	0.126
	*t_xbi_*	Idle	318.7	N/A
	*t_xbs_*	Sleep	N/A	319.874

## References

[1] Yick J., Mukherjee B., Ghosal D. (2008). Wireless sensor network survey. Comput. Netw..

[2] Karagiannis A., Vouyioukas D., Constantinou P. Energy consumption measurement and analysis in wireless sensor networks for biomedical applications.

[3] Ruiz-Garcia L., Lunadei L., Barreiro P., Robla I. (2009). A review of wireless sensor technologies and applications in agriculture and food industry: State of the art and current trends. Sensors.

[4] Akyildiz I.F., Su W., Sankarasubramaniam Y., Cayirci E. (2002). Wireless sensor networks: A survey. Comput. Netw..

[5] Baca A., Kornfeind P., Preuschl E., Bichler S., Tampier M., Novatchkov H. (2010). A Server-Based Mobile Coaching System. Sensors.

[6] Shin H.-Y., Un F.-L., Huang K.-W. A Sensor-Based Tracking System for Cyclist Group.

[7] Marin-Perianu R., Marin-Perianu M., Havinga P., Begg T.R., Palaniswami M., Rouffet D. (2013). A performance analysis of a wireless body-area network monitoring system for professional cycling. Pers. Ubiquit Comput..

[8] Zulkifli N., Harun F.C., Azahar N. XBee wireless sensor networks for Heart Rate Monitoring in sport training.

[9] Sendra S., Lloret J., García M., Toledo J.F. (2011). Power saving and energy optimization techniques for Wireless Sensor Networks. J. Commun..

[10] Shebli F., Dayoub I., Rouvaen J. Minimizing energy consumption within a wireless sensor network.

[11] Yan R., Sun H., Qian Y. (2013). Energy-Aware Sensor Node Design with Its Application in Wireless Sensor Networks. IEEE Trans. Instrum. Meas..

[12] Zhen C., Liu W., Liu Y., Yan A. (2014). Energy-Efficient Sleep/Wake Scheduling for Acoustic Localization Wireless Sensor Network Node. Int. J. Distrib. Sens. N..

[13] Azevedo J., Santos F., Rodrigues M., Aguiar L. (2014). Sleeping ZigBee networks at the application layer. IET Wirel. Sens. Syst..

[14] Cortés-Sánchez J., Velázquez-Ramírez A., Lucas-Bravo A., Rivero-Angeles M.E., Salinas-Reyes V.A. (2014). On the use of electromagnetic waves as means of power supply in wireless sensor networks. EURASIP J. Wirel. Comm. Netw..

[15] Liu Z., Yang X., Yang S., McCann J. (2013). Efficiency-Aware: Maximizing Energy Utilization for Sensor Nodes Using Photovoltaic-Supercapacitor Energy Systems. Int. J. Distrib. Sens. Netw..

[16] Stojcev M.K., Kosanovic M.R., Golubovic L.R. Power management and energy harvesting techniques for wireless sensor nodes.

[17] Zhou G., Huang L., Li W., Zhu Z. (2014). Harvesting Ambient Environmental Energy for Wireless Sensor Networks: A Survey. J. Sens..

[18] Cryns J.W., Hatchell B.K., Santiago-Rojas E., Silvers K.L. (2013). Experimental Analysis of a Piezoelectric Energy Harvesting System for Harmonic, Random, and Sine on Random Vibration. Adv. Acoust. Vib..

[19] Chao P.-P. (2011). Energy harvesting electronics for vibratory devices in self-powered sensors. IEEE Sens. J..

[20] Barratt P. (2008). SRM TorqueAnalysisof Standing Starts in Track Cycling (P85). The Engineering of Sport 7.

[21] Marr D. (1989). Bicycle Gearing: A Practical Guide.

[22] SRM http://www.srm.de.

[23] Polar http://www.polar.com.

[24] Monk S. (2012). Programming Arduino: Getting Started with Sketches.

[25] Al-Kuwari A.M., Ortega-Sanchez C., Sharif A., Potdar V. User friendly smart home infrastructure: BeeHouse.

[26] Bayle J. (2013). First Contact with C. C Programming for Arduino.

[27] Bell C. (2013). Tiny Talking Modules: An Introduction to XBee Wireless Modules. Beginning Sensor Networks with Arduino and Raspberry Pi.

[28] Di Francesco M., Anastasi G., Conti M., Das S.K., Neri V. (2011). Reliability and Energy-Efficiency in IEEE 802.15.4/ZigBee Sensor Networks:An Adaptive and Cross-Layer Approach. IEEE J. Sel. Areas Commun..

[29] Hebel M., Bricker G. Getting Started with XBee RF Modules: A Tutorial for BASIC Stamp and Propeller Microcontrollers. http://www.parallax.com/downloads/getting-started-xbee-rf-modules-text.

[30] XCTU Software. http://www.digi.com/support/productdetail?pid=3352&osvid=57type=utilities.

[31] Men's Team Pursuit Gold Final-Great Britain, Austrial-2013 UCI World Track Championship. http://www.youtube.com/watch?v=QExadv_WxKw.

[32] Casilari E., Cano-García J.M., Campos-Garrido G. (2010). Modeling of Current Consumption in 802.15.4/ZigBee Sensor Motes. Sensors.

[33] Currell D. Track Cycling—An Introduction What A Roadie Needs to Know to Start Racing on the Velodrome. www.ridethetrack.com/pdf/trackracing_intro.pdf.

[34] ATMEL 8-Bit AVR Microcontroller with 4/8/16/32K bytes in System Programmable Flash. http://www.atmel.com/Images/doc8161.pdf.

[35] Rahman M.N., Matin M. (2011). Efficient algorithm for prolonging network lifetime of wireless sensor networks. Tsinghua Sci. Technol..

[36] Efendi A.M., Oh S., Negara A.F.P., Choi D. (2013). Battery-Less 6LoWPAN-Based Wireless Home Automation by Use of Energy Harvesting. Int. J. Distrib. Sens. Netw..

[37] Parallax Microcontroller Data Acquisition for Excel. http://www.parallax.com.

[38] Gardner A.S., Martin D.T., Barras M., Jenkins D.G., Hahn A.G. (2005). Power output demands of elite track sprint cycling. Int. J. Perform. Anal. Sport.

[39] Zhan A., Chang M., Chen Y., Terzis A. Accurate caloric expenditure of bicyclists using cellphones.

[40] Burchfield T.R., Venkatesan S., Weiner D. Maximizing Throughput in ZigBee Wireless Networks through Analysis, Simulations and Implementations. http://www.mobiusconsulting.com/papers/ZigBee_Throughput.pdf.

[41] Moravek P., Komosny D., Simek M., Mraz L. Energy demands of 802.15.4/ZigBee communication with IRIS sensor motes.

[42] Zhou H.-Y., Luo D.-Y., Gao Y., Zuo D.-C. (2011). Modeling of node energy consumption for wireless sensor networks. Wirel. Sens. Netw..

[43] Wentao D., Wenfeng L., Fan Z. Consensus algorithm for energy consumption of wireless sensor networks.

[44] Bachmann C., Ashouei M., Pop V., Vidojkovic M., Groot H.D., Gyselinckx B. (2012). Low-power wireless sensor nodes for ubiquitous long-term biomedical signal monitoring. IEEE Commun. Mag..

[45] Wang W.S., O'Keeffe R., Wang N., Hayes M., O'Flynn B., Ó Mathúna S.C. Practical wireless sensor networks power consumption metrics for building energy management applications.

[46] Bluetooth Low Energy and 2.4GHz Proprietary Multiprotocol Soc. http://www.nordicsemi.com/eng/Products/Bluetooth-R-low-energy.

[47] Imtiaz S.A., Casson A.J., Rodriguez-Villegas E. (2014). Compression in Wearable Sensor Nodes: Impacts of Node Topology. IEEE Trans. Biomed. Eng..

[48] Jurdak R., Ruzzelli A.G., O'Hare G.M.P. (2010). Radio Sleep Mode Optimization in Wireless Sensor Networks. IEEE Trans. Mobile Comput..

[49] Texas Instruments, CC2520 (Second generation 2.4 GHz ZigBee/IEEE 802.15.4 RF transceiver). http://focus.ti.com/docs/prod/folders/print/cc250.html.

[50] Qu T., Chen X., Zhang L., Xia C., Dai S. Zigbee wirelessly network module design based on CC2480.

[51] Faludi R. (2011). Building Wireless Sensor Networks.

